# The gut’s hidden arsenal: A genomics-guided atlas of class II bacteriocins

**DOI:** 10.1016/j.xgen.2025.101064

**Published:** 2025-11-12

**Authors:** Tianang Leng, Cesar de la Fuente-Nunez

**Affiliations:** 1 Machine Biology Group, Departments of Psychiatry and Microbiology, Institute for Biomedical Informatics, Institute for Translational Medicine and Therapeutics, Perelman School of Medicine, University of Pennsylvania, Philadelphia, PA, USA; 2 Departments of Bioengineering and Chemical and Biomolecular Engineering, School of Engineering and Applied Science, University of Pennsylvania, Philadelphia, PA, USA; 3 Department of Chemistry, School of Arts and Sciences, University of Pennsylvania, Philadelphia, PA, USA; 4 Penn Institute for Computational Science, University of Pennsylvania, Philadelphia, PA, USA

## Abstract

Unmodified class II bacteriocins promise precision antimicrobials that spare bystander microbes. Zhang and colleagues introduce IIBacFinder, a genomics-guided pipeline that detects precursor and context genes with a curated pHMM library, infers leader-peptide cleavage, and triages candidates by meta-omics signals. The authors apply it across bacterial genomes, including an atlas of ∼280,000 human-gut genomes, and recover a vast reservoir of narrow-spectrum peptides and prioritize gut-resident candidates for synthesis. Of the 26 synthesized, 16 display activity *in vitro*, largely via membrane perturbation and with additive effects alongside vancomycin, while *ex vivo* assays show minimal compositional disruption of fecal communities compared with antibiotic controls. These results position unmodified class II bacteriocins as tractable, microbiome-sparing agents and illustrate how genome-scale mining coupled to meta-omics can bridge sequence to function in complex ecosystems.

## Main text

Antimicrobial peptides (AMPs) are a ubiquitous innate defense strategy across all domains of life,^1^ with broad activities against bacteria, fungi, viruses, and parasites. These small, ribosomally encoded peptides often have immunomodulatory effects and can synergize with conventional antibiotics. However, a “potency-first” discovery approach risks ecological collateral damage: broad-spectrum peptides and small molecule antibiotics tend to wipe out commensal microbes and destabilize microbial communities. By contrast, bacteriocins—ribosomally synthesized peptides produced by bacteria—offer a precision alternative. Unlike antibiotics, most bacteriocins have a narrow spectrum of activity, targeting a specific pathogen while sparing the rest of the microbiota. In particular, unmodified class II bacteriocins are heat stable and often narrow spectrum. They come in several subclasses (class IIa, pediocin-like; IIb, two-peptides; IIc, circular peptides; and IId, linear non-pediocin-like).^2^^,^^3^ Their simpler chemistry makes them easier to synthesize and engineer, and in some cases, they are linked to host-friendly functions. This motivates genome-guided mining beyond motif-only detection tools such as BAGEL4 or broad ribosomally synthesized and post-translationally modified peptide (RiPP) detectors such as antiSMASH.

**Figure 1:**
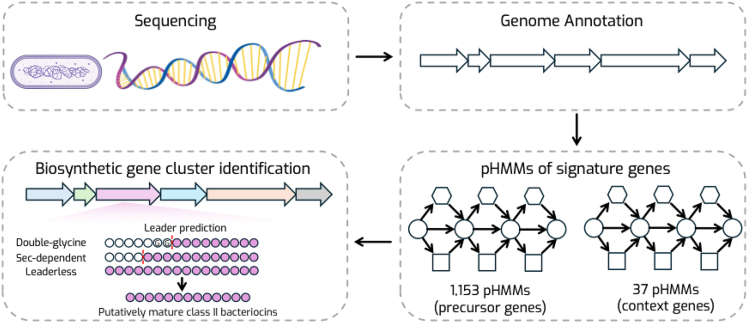
Schematic overview of the genome mining procedure in IIBacFinder The IIBacFinder workflow comprises four main modules: (1) sequencing—genomic DNA from isolated or publicly available bacterial strains is sequenced and assembled using short- and/or long-read platforms. (2) Genome annotation—the assembled genomes are structurally and functionally annotated to identify open reading frames and gene boundaries. (3) pHMMs of signature genes—curated pHMMs are used to detect signature genes across genomes, including 1,153 pHMMs for precursor peptides and 37 pHMMs for context. (4) Biosynthetic gene cluster (BGC) identification—predicted hits are integrated to reconstruct bacteriocin biosynthetic gene clusters, followed by leader peptide prediction that distinguishes and identifies cleavage motifs of different precursors. This figure was partially created in BioRender. De La Fuente-Nunez, C. (2025) https://BioRender.com/8hgb3a8

Zhang and colleagues respond to this challenge with IIBacFinder,^4^ a specialized computational pipeline for unmodified class II bacteriocins. The pipeline integrates detection of both precursor peptide genes and neighboring context genes (transporters, immunity proteins, and peptidases) using a curated library of profile hidden Markov models (pHMMs). Next, it predicts cleavage of leader peptides using an adapted version of NLPPrecursor for peptides with double-glycine leaders and SignalP 6.0 for Sec-dependent leaders. Finally, the tool filters the candidate mature cores against known AMP databases to assess novelty (Figure 1). In benchmarking on 142 genomes harboring 228 characterized class II bacteriocins, IIBacFinder recovered all 228 known peptides (including 32 held out from training), whereas BAGEL4 and antiSMASH detected only 64.9% and 55.7% of them, respectively. By explicitly encoding biosynthetic logic (leader peptides, transporters, and peptidases) into the search, the pipeline overcomes the common failure modes of precursor-only tools.

The authors then explore the broader biosynthetic landscape of unmodified bacteriocins. Applying IIBacFinder to 257,997 RefSeq genomes spanning 56 bacterial phyla, they detect 540,587 candidate precursor peptides, of which 481,654 pass stringent post-processing filters. Strikingly, the potential to produce class II bacteriocins is found across the bacterial tree, including in Gram-negative phyla (for example, *Pseudomonadota* and *Bacteroidota*) that have historically been underrepresented. Most predicted bacteriocins show habitat specificity, echoing patterns seen for other microbiome-derived AMPs. Together, these data argue that unmodified bacteriocins are not a Gram-positive peculiarity but rather a pervasive feature of bacterial life.

Focusing on the human gut, Zhang et al. apply IIBacFinder to ∼280,000 gut-derived genomes and identify 82,806 high-confidence precursor peptides, clustering into 6,238 unique mature sequences. These gut-encoded bacteriocins come from diverse taxa—*Clostridia*, *Bacilli*, and *Bacteroidia*—dominated by abundance, while *Streptococcus* contributes outsized sequence diversity. Comparison to existing bacteriocin databases shows that most gut candidates are novel. Furthermore, analysis of 724 gut metatranscriptomes reveals active transcription of roughly one-third of the candidate sequences: individual hosts simultaneously express tens to hundreds of distinct bacteriocins. These findings suggest that the healthy gut harbors a constitutive, multipronged defensive arsenal of unmodified bacteriocins that is personalized to each host yet prevalent across the population.

To select the most promising candidates for experimentation, the authors prioritize peptides predicted to be tractable and physiologically relevant. They construct a non-redundant reference set of 32,735 bacteriocin clusters by scanning ∼700,000 genomes from human, animal, and environmental sources. Each cluster is scored across 1,901 metagenomes and 724 metatranscriptomes from healthy donors. Ranking balances prevalence and abundance with ecological footprint: clusters showing negative associations with particular taxa or correlations with increased community diversity are favored on the hypothesis that an active bacteriocin will leave detectable signals in community profiles. From this ranking, the authors select 40 high-priority clusters for validation. They synthesize 26 representative peptides and test them *in vitro*: 16 of the 26 (≈70%) inhibit at least one indicator strain, predominantly Gram-positive targets, with minimum inhibitory concentrations in the single-digit to tens of μg/mL range. Mode-of-action assays show that several candidates permeabilize the bacterial membrane, while others cause membrane depolarization without overt leakage—indicating a spectrum of killing mechanisms. Checkerboard assays reveal additive interactions with vancomycin (fractional inhibitory concentration index ≈ 1), suggesting complementary mechanisms rather than true synergy.

Crucially, the authors test the peptides’ compatibility with complex communities. In fecal-derived *ex vivo* cultures, three of the most active peptides (plus a weakly active control) are applied at 1 or 100 μg/mL. Unlike 100 μg/mL vancomycin, which induces pronounced dysbiosis, each bacteriocin produces negligible changes in global community composition and α-diversity over 48 h. Even at the highest dose, only two peptides alter the abundance of a limited set of species. Extended exposure (six days) likewise shows minimal divergence from DMSO controls in bacteriocin-treated cultures—no cumulative drift—whereas vancomycin drives sustained shifts. These data strongly support the authors’ central premise: narrow-spectrum bacteriocins can achieve antibacterial activity with minimal impact on the broader microbiome.

The study includes two additional validation fronts. First, the authors test generality beyond the gut by synthesizing a panel of predicted bacteriocins from diverse phyla. Most of these peptides exhibit antimicrobial activity *in vitro*, with several reaching sub-μg/mL minimal inhibition concentrations (MICs), demonstrating that IIBacFinder generalizes across habitats. Second, a comparative physicochemical analysis highlights how class II bacteriocins differ from generic AMP libraries. The discovered bacteriocins tend to be longer and carry a lower net positive charge than typical AMPs do. The subset with Sec-dependent leaders also shows a higher predicted protein-binding propensity (Boman index). These features imply distinct membrane-interaction and target-binding modalities, which likely underlie the bacteriocins’ narrow spectra and help spare bystanders.

Altogether, Zhang et al. deliver a rigorous genome-first blueprint for discovering microbiome-sparing antimicrobials. By explicitly encoding biosynthetic context into peptide detection, leveraging large-scale meta-omic data to identify the most relevant candidates, and validating activity with careful community-level assays, they bridge the gap from sequence to function *in situ*. Looking forward, coupling IIBacFinder to high-throughput cell-free expression systems and activity screens could accelerate discovery. Systematic mutagenesis of leader and core peptide motifs could reveal determinants of stability, secretion, and target specificity. Finally, assembling rational cocktails—whether between complementary bacteriocins or in combination with conventional antibiotics—may further broaden antimicrobial coverage while preserving microbial community structure.

Indeed, this work exemplifies the broader surge of AI-/machine learning (ML)-driven peptide discovery. ML platforms have recently accelerated AMP exploration across many fronts, from the use of evolutionary computation to design new synthetic peptides, such as *guavanin-2*,^5^ to advanced generative models and reinforcement learning.^6^ For example, diffusion-based frameworks can condition peptide generation on pathogen features,^7^ and even deep-learning “molecular de-extinction” pipelines mine ancient proteomes for hidden antimicrobial motifs.^8^ These AI-powered strategies complement IIBacFinder, together heralding an emerging paradigm of computational antibiotic discovery that bridges sequence databases to *in vivo* efficacy.

## Acknowledgments

C.d.l.F.-N. holds a Presidential Professorship at the University of Pennsylvania.

## Declaration of interests

C.d.l.F.-N. is a co-founder of and scientific advisor to Peptaris, Inc.; provides consulting services to Invaio Sciences; and is a member of the scientific advisory boards of Nowture S.L., Peptidus, European Biotech Venture Builder, the Peptide Drug Hunting Consortium (PDHC), ePhective Therapeutics Inc., and Phare Bio.

## Declaration of generative AI and AI-assisted technologies in the writing process

During the preparation of this manuscript, the authors used the generative AI tool ChatGPT (OpenAI) to improve readability and language clarity. After using this tool, the authors carefully reviewed and edited all content and take full responsibility for the accuracy and integrity of the publication.
